# Exploring the course of functional somatic symptoms (FSS) from pre- to late adolescence and associated internalizing psychopathology – an observational cohort-study

**DOI:** 10.1186/s12888-024-05937-3

**Published:** 2024-07-08

**Authors:** Lina Münker, Martin Køster Rimvall, Lisbeth Frostholm, Eva Ørnbøl, Kaare Bro Wellnitz, Pia Jeppesen, Judith Gerarda Maria Rosmalen, Charlotte Ulrikka Rask

**Affiliations:** 1 Department of Child and Adolescent Psychiatry, Aarhus University Hospital Psychiatry, Psychiatry, Aarhus, Denmark; 2 Department of Functional Disorders and Psychosomatics, Aarhus University Hospital, Aarhus, Denmark; 3 Department of Clinical Medicine, Aarhus University, Aarhus, Denmark; 4 Department of Child and Adolescent Psychiatry, Copenhagen University Hospital - Psychiatry Region Zealand, Roskilde, Denmark; 5 Child and Adolescent Mental Health Centre, Copenhagen University Hospital - Mental Health Services CPH, Copenhagen, Denmark; 6 Department of Clinical Medicine, Faculty of Health and Medical Sciences, University of Copenhagen, Copenhagen, Denmark; 7 Departments of Psychiatry and Internal medicine, University of Groningen, University Medical Center Groningen, Groningen, Netherlands

**Keywords:** Functional Somatic Symptoms, Anxiety, Depression

## Abstract

**Background:**

Functional somatic symptoms (FSS), which commonly cannot be attributed to well-defined organic pathology, often co-occur with internalizing psychopathology and fluctuate throughout different life stages. We examined FSS courses throughout adolescence, and the association between preadolescent FSS, FSS severity and internalizing psychopathology at late adolescence.

**Methods:**

Data from the Copenhagen Child Cohort (CCC2000) were utilized from assessments at ages 11–12 years (preadolescence; T0) and 16–17 years (late adolescence; T1). Self-report questionnaire and interview data on FSS, internalizing psychopathology, chronic medical conditions, and sociodemographic data from Danish national registers were available for 1285 youths. FSS courses were categorized into persistent (high FSS at T0 & T1), remission (high FSS only at T0), incident (high FSS only at T1) or no FSS (no FSS at T0 & T1). Multiple linear and multinomial logistic regressions were conducted to investigate the FSS/psychopathology association.

**Results:**

1.8% of adolescents fell into the persistent FSS course group throughout adolescence. Higher preadolescent FSS predicted FSS (*b* = 0.07, *p* < .001), anxiety (*b* = 0.05, *p* < .001) and depression (*b* = 0.06, *p* < .001) at age 16/17, even after controlling for sex, parental education, a chronic medical condition and internalizing psychopathology in preadolescence. Persistent, incident, and remittent FSS courses were associated with significantly higher mean levels of anxiety and depression compared to the reference group (no FSS).

**Conclusions:**

FSS during pre- and late adolescence might increase and co-occur with anxiety and depression throughout adolescence, potentially due to shared underlying risk factors and processes.

**Supplementary Information:**

The online version contains supplementary material available at 10.1186/s12888-024-05937-3.

## Introduction

Somatic symptoms that commonly cannot be attributed to well-defined organic pathology are often referred to as functional somatic symptoms (FSS). Common explanatory models of FSS emphasize a complex underlying etiology [1], with changes in bodily functioning rather than structure [2]. Yet, presence of medical comorbidity is well recognized, emphasizing that FSS can occur along with physical diseases [3]. FSS can be regarded as an indicator of low mental well-being with different expressions of bodily distress, i.e. various physical responses to prolonged stress in susceptible individuals [3, 4]. FSS are already common in childhood or adolescence, affecting approximately 25–30% [5, 6]. Whereas the symptoms tend to present equally among boys and girls in early childhood (i.e. at age 5–7), they are more common among females than males in adolescence [7, 8]. FSS can affect any bodily system, where abdominal pain represents most frequently in children, and headaches, muscular soreness or fatigue being more common in adolescents [9, 10]. A mono-symptomatic (i.e. a single prominent symptom) presentation is more usual in young children [11, 12].

Only few lines of research have investigated developmental courses of FSS across early life stages over time. Different symptom courses or trajectories have been reported, suggesting that for some young people, symptoms seem to persist and are associated with impairment [13–15]. In these longitudinal studies, Mulvaney et al. reported their long-term risk group for symptoms to be approximately 14% during youth in pediatric patients [13], with similar percentages from Nummi et al. in a general population study, i.e. approximately 17% in the high symptom load trajectory from late adolescence to middle adulthood [14]. A lower number of approximately 4% of persistent FSS from the ages 11–16 among 2210 adolescents was reported in the prospective Dutch cohort study TRacking Adolescents’ Individual Lives Survey (TRAILS) [16]. Further, the longitudinal, general population Zurich Epidemiological Study of Child and Adolescent Psychopathology (ZESCAP) (lasting from late childhood to early adulthood), suggested lower numbers of approximately 1 to 9% when looking at individual symptom trajectories [15]. For others, symptoms improve or remit [13, 14].

Adolescence represents a potentially vulnerable phase, characterized by many physical and psychosocial changes [17] during which psychological problems such as internalizing problems frequently emerge [18–21]. Internalizing psychopathology, like anxiety and depression, often accompanies somatic symptoms already early in life, and over time [16, 22–36]. The directionality of the association between FSS and anxiety and depression is yet less understood [22, 37]. FSS during childhood and adolescence could contribute to the risk for later internalizing psychopathology [24, 29, 38–40], but somatic symptoms at an earlier stage might also contribute to somatic symptom severity later on [41]. Potential explanations for the association between FSS and internalizing psychopathology might be shared vulnerability factors [22, 42–44], where symptom expressions are understood as independent but not conceptually distinct expressions of distress [22, 45–47]. Contemporary conceptual frameworks of psychopathology, such as the Hierarchical Taxonomy Of Psychopathology (HiTOP) model [48, 49], emphasize associations between different types of symptomatology. According to this model, FSS and internalizing symptoms are proposed to be linked within an ‘Emotional dysfunction superspectrum’, due to their suspected underlying shared risk factors and pathological mechanisms in the biological (i.e. genetic vulnerability, neural biomarkers), environmental (i.e. childhood maltreatment, life stress) and psychological (i.e. cognitive and affective difficulties, childhood temperament) domains. Generalizability of the model is suggested to be tested across a broader age range [50].

Given that recovery from conditions characterized by FSS may become more difficult with increasing age, early identification and intervention is essential to prevent adverse long-term health outcomes [51, 52]. Still, epidemiological research on the course of FSS, and how these symptoms are associated with internalizing psychopathology during adolescence, is scarce. Therefore, we need to advance knowledge not only on FSS courses but also their association with anxiety and depression during adolescence to understand FSS development in a broader perspective together with accompanying psychopathology over time [4]. This will ultimately contribute to a better understanding of the dynamic interplay between these symptom domains and what determines the trajectories of persistent but also different symptom expressions over time. In the current study, our aims were fourfold: we investigated objective (1) FSS courses from pre- (i.e. at age 11–12) to late (i.e. at age 16–17) adolescence, objective (2) the association between preadolescent FSS and FSS presence, anxiety and depression symptoms in late adolescence, while accounting for internalizing psychopathology in preadolescence, objective (3) the association between preadolescent FSS and FSS severity in late adolescence and objective (4) how different FSS courses throughout adolescence are associated with anxiety and depression in late adolescence.

## Materials and methods

### Study population

This study was pre-registered on the Open Science Framework platform (Registration DOI: 10.17605/OSF.IO/G42J6). The study is based on the Copenhagen Child Cohort (CCC2000) [53], where 6090 children born in the year 2000 in the former Copenhagen County, Denmark, were followed over time, from infancy to late adolescence. The original cohort was representative of the Danish child population with respect to perinatal and sociodemographic characteristics [53]. We utilized data from the 11-12-years (CC11/12, baseline: T0) and 16-17-years (CC16/17, follow-up: T1) assessment waves. Cohort members of the CC11/12 were invited by postal letters, while contacted through an established governmental e-mail system at CC16/17, and were asked to fill in online questionnaires at both assessment waves [53]. The duration of responding to the online questionnaires took approximately 45–90 min at T0 and 30–60 min at T1.

### Functional somatic symptoms

At age 11/12, FSS were assessed by the Children’s Somatic Symptoms Inventory (CSSI, formerly Children’s Somatization Inventory (CSI)) [54]. The children were asked to rate 24 somatic symptoms (e.g. ‘Headache’; ‘Faintness or dizziness’) according to the instruction ‘How much were you bothered by (symptom)?’ in the last two weeks on a 5-point rating scale from ‘Not at all’ (0) to ‘A whole lot’ (4) (total sum score range 0–96, higher scores corresponding to higher self-reported symptom presence and severity). The CSI-24/CSSI-24 was originally developed to standardize assessment of bothersome unspecific somatic symptoms, conceptualized as FSS, in pediatric patients. Subsequently the measure has been extensively evaluated and shown good psychometric properties in assessing FSS in both community and clinical samples across various cultural contexts [55–57]. Further, the CSSI-24 has been found useful for the purpose of following somatic symptom development over time as well as for monitoring treatment response in clinical populations with an established functional somatic syndrome diagnosis [55].

At age 16/17, the revised Bodily Distress Syndrome (BDS) 25-checklist was used to assess FSS according to four symptom clusters: cardio-pulmonary, gastro-intestinal, musculoskeletal, and general symptoms [58]. The BDS-checklist has displayed good psychometric properties in Danish adult and adolescent populations [58–60]. Participants were asked to respond to ‘Within the past 12 months, to what extent have you been bothered by’ on 25 physical symptoms, along a 5-point rating scale ranging from ‘Not at all’ (0) to ‘A lot’ (4). A total sum score of FSS was calculated (score range 0-100; higher ratings corresponding to higher self-reported symptom presence and severity).

In order to explore FSS courses from pre- to late adolescence (objective 1), FSS sum scores at both respective follow-up moments were dichotomized into high (top 10%; high symptom load) and low (bottom 90%; no/low symptom load) scores on basis of the entire participant population, as done in former CCC2000 studies [61, 62]. Subsequent FSS courses were defined by four groups: no FSS at any time (no/low FSS at T0 and T1), remission (high FSS only at T0), incident (high FSS only at T1) and persistent (high FSS at T0 and T1).

In order to investigate the association between preadolescent FSS and FSS, anxiety and depression at late adolescence (objective 2), the total FSS sum score at 11–12 was used as a continuous independent variable. Lastly, we investigated the association between preadolescent FSS and FSS severity in late adolescence (objective 3) where FSS severity at CC16/17 was determined by results from our previously published Latent Class Analysis using conditional probabilities of fulfilling specified symptom criteria to define the following classes: (1) probable no to mild FSS (sub-group with no significant somatic symptoms in any symptom cluster), (2) probable moderate, single-organ FSS (sub-group with significant symptoms in one to two symptom clusters) and (3) probable severe, multi-organ FSS (sub-group with significant symptoms in multiple symptom clusters) [58].

### Internalizing psychopathology

At age 11–12, self-reported internalizing psychopathology was assessed using the ‘emotional problems’ subscale of The Strengths and Difficulties Questionnaire (SDQ) [63], which includes 5 items. The following instructions were provided: ‘For each item, please mark the box for ‘Not True’ (0), ‘Somewhat True’ (1) or ‘Certainly True’ (2)’ (example item: ‘I am often unhappy, depressed or tearful’). We excluded one item (Item 3: *‘Often complains of headaches, stomach-aches or sickness…*’) due to a thematic overlap with somatic symptoms already assessed by the CSSI-24. Thus, the total sum score was based on the remaining four items (score range 0–8; higher ratings indicating greater level of self-reported internalizing psychopathology), which provided moderate internal validity (Cronbach’s α = .66) and is comparable to including item 3 (Cronbach’s α = .68).

At age 16–17, depression and anxiety were assessed in separate questionnaires. Depression was assessed using the Mood and Feelings Questionnaire (MFQ) [64] including 33 items. The adolescent was asked: ‘How have you been within the past 2 weeks’ (example item: ‘I felt miserable and unhappy.’). Response options ranged from ‘Not true’ (0) to ‘True’ (2). We calculated a total sum score (score range 0–66, higher rating indicating more self-reported depressive symptoms; cut-off indication for potential presence of depression: ≥27 [65]). To assess anxiety, the Spence Children’s Anxiety Scale (SCAS) [66], including 44 items, was employed. Potential anxiety evoking situations are presented to the adolescent together with how frequent the anxiety evoking situations occur, along a 4-point rating scale from ‘Never’ (0) to ‘Always’ (3). The filler items were included in an effort to hide the true purpose of the questionnaire, and thereby reduce potential response bias, as these items were unrelated to the anxiety construct. Accordingly, these items are excluded in a total score based on 38 items (score range 0-114; higher ratings indicating higher self-reported anxiety levels; cut-off indication for elevated anxiety: T-score ≥ 60, depending on age and sex of the child [67]). Both MFQ and SCAS have been validated in Danish samples with comparable age groups [64, 68].

### Socio-demographic variables

Child sex assigned at birth (i.e. ‘male’/‘female’) was obtained from the Danish Civil Registry [69], and was used as covariate in the analyses to adjust for potential sex-specific differences in FSS development and expression in adolescence [58, 70]. Parental education at age 11/12 was derived from the Integrated Labor Market Registry [71], and categorized according to either parent’s highest education level, i.e. (1) Primary school education (up to grade 9) and/or High School, (2) Short Traineeship, or (3) Long Traineeship/University education, and was used as covariate in the analyses to adjust as a proxy for familial adversity and its potential impact on FSS [72].

### Chronic medical conditions

Presence of a chronic medical condition at age 11–12 was derived from the parent-report Soma Assessment Interview (SAI) [73], according to an a priori list of well-defined medical conditions. According to the instruction, ‘Within the past 12 months, has your child suffered from any of these physical illnesses or handicaps?‘, the parent is asked whether a physician has diagnosed any of a list of chronic medical conditions (‘asthma’, ‘heart disease’, ‘epilepsy’, ‘rheumatic disease’, ‘kidney disease’, ‘diabetes’, ‘severe vision or hearing problem or total blind- or deafness’, ‘disorders that affect the function of nerves and muscles, i.e. cerebral palsy, spina bifida, muscular dystrophy, specify…’, ‘other serious physical diseases or disabilities, specify…’) in their child, with a ‘Yes’/‘No’ response option. A ‘Yes’ response to any of the listed medical conditions was considered presence of a chronic medical condition. Chronic medical condition was used as covariate in the analyses, as it is recognized that FSS can co-occur with a somatic disease [3]. However, the presence of such a condition may also partly explain the reported somatic symptoms and should therefore be adjusted for.

### Statistical analyses

Due to the described potential conceptual overlap between FSS and internalizing psychopathology in existing literature [22], we performed simple correlation analyses within and across time points to check to what extent different concepts were indeed associated. In order to explore objective 2, i.e. the association between preadolescent FSS with FSS and internalizing psychopathology in late adolescence, we conducted multiple linear regressions. These were performed separately for each outcome variable at T1: (1) FSS, (2) Anxiety, and (3) Depression and in a stepwise manner according to 4 models with increasing adjustment for chosen covariates as follows: Model (1) crude model (FSS at T0 only), Model (2) sex, parental education (FSS, and sex and parental education as covariates at T0), Model (3) concurrent chronic medical condition (FSS, and sex, parental education and concurrent chronic medical condition as covariates at T0), Model (4) internalizing psychopathology (FSS, and sex, parental education, concurrent chronic medical condition and internalizing psychopathology as covariates at T0). The addition of a measure of internalizing psychopathology at age 11–12 in the last model was to adjust for those with already existing emotional problems at the earlier age. Model assumptions respective to linear regression models (i.e., homoscedastic and normally distributed residuals and linearity of predictors) were evaluated on visual inspection of residual plots as well as plots of natural cubic splines. On basis of assumption checks, we decided to transform all continuous outcome variables (i.e. FSS, depression and anxiety), using the square root transformation.

To investigate the association between FSS at T0 and FSS severity (i.e. objective 3) i.e. (1) probable no to mild FSS, (2) probable moderate, single-organ FSS and (3) probable severe, multi-organ FSS at T1, ordinal logistic regression was planned. However, due to violation of the assumption of proportional odds, multinomial logistic regression was performed instead. Finally, to investigate the association between the FSS courses and internalizing psychopathology at T1 (objective 4), simple linear regression was performed, using the FSS course variable as a single categorical independent variable on anxiety and depression at T1.

We decided to only apply the crude model with no further adjustments (Model 1) in the analyses corresponding to objective 3 due to power issues with the small sample size of the FSS severity group probable severe, multi-organ FSS as outcome variable at T1 (*n* = 74) (objective 3). Regarding objective 4, the small group of persistent FSS with *n* = 23 would mean a wider confidence interval in comparison to the other FSS courses, which would be more pronounced with more adjustments, potentially hampering more accurate interpretation. Therefore, we decided to apply only the crude model also in this analysis. We conducted all analyses on the digital Denmark Statistics server, using STATA [74]. A two-sided *p*-value of 0.05 was used as inference criteria.

## Results

### Attrition

At CC11/12, 2349 cohort members responded to online questionnaires, with complete FSS data for *n* = 1890. These children were characterized by a higher proportion of females (52.49% vs. 46.96%) and parents with higher level of education (24.39% vs. 15.48%) (*X*^2^ tests, both *p* < .01). At CC16/17, 2614 cohort members responded to online questionnaires with complete FSS data for *n* = 2542. The final study population, i.e. those participants contributing to assessments of self-reported FSS at both CC11/12 and CC16/17, comprised of *N* = 1285. Participants with data from both assessments were more often female and had parents with a higher level of education, whereas there were no statistical significant differences on measures of FSS, internalizing psychopathology and presence of a chronic medical condition at age 11–12 compared to participants lost to follow-up (see Appendix 1).

### Sample characteristics and FSS courses

Table 1 summarizes the distribution of measured socio-demographics and reported chronic medical condition at T0 for the four FSS courses. The total sample, comprising of 1285 participants, composed of more females (% not shown due to regulations of data protection of the Denmark Statistics server). The majority of participants had parents with a short traineeship as highest education level (67.3%), with the smallest proportion with parents with a primary school/high school as highest education level (5.2%). Out of the total sample, 13.2% had a chronic medical condition at age 11–12.

**Fig. 1:**
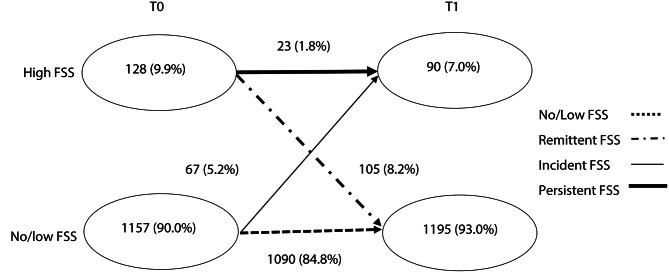
FSS courses from pre- (T0, age 11–12) to late (T1, age 16–17) adolescence (*N* = 1285) *Note*: Abbreviations: FSS = Functional Somatic Symptoms; FSS at T0 and T1 was dichotomized into high (top 10% score; high symptom load) and low scores (bottom 90% score; no/low symptom load) on basis of the entire participant population at the respective follow-up moment

Overall, more females than males reported high FSS. This tendency was especially pronounced in the incident and persistent FSS course group. Furthermore, relatively more participants with persistent symptoms reported the presence of a chronic medical condition at age 11–12 and had at least one parent with a higher education level. Figure 1 displays group sizes of the participants falling in the different categorized FSS courses from pre- (T0) to late (T1) adolescence. In total, 9.9% at T0 and 7.0% at T1, respectively, reported high symptom load. The majority of participants reported low FSS at both assessments (84.8%), while the smallest percentage of participants reported persisting high symptoms (1.8%). Slightly more participants were placed into the remittent FSS course (8.2%) than into the FSS course with incident FSS (5.2%).

**Table 1:** Distribution of covariates (sociodemographic variables and presence of a chronic medical condition) at T0 (age 11–12) according to FSS courses (*N* = 1285)

	No/Low FSS *n* = 1090	Remittent FSS *n* = 105	Incident FSS *n* = 67	Persistent FSS *n* = 23	
	*n* (%**)				
Sex					
Male	520 (47.7)	43 (40.9)	8 (11.9)	-*	
Female	570 (52.3)	62 (59.1)	59 (88.1)	-*	
Highest parental education; *Missingn* = *8 (0.6%)*					
Primary school/ High School Short Traineeship Long Traineeship/University	53 (4.9) 721 (66.2) 309 (28.4)	8 (7.6) 77 (73.3) 19 (18.1)	6 (8.9) 52 (77.6) 9 (13.4)	0 (0.0) 15 (65.2) 8 (34.8)	
Chronic medical condition; *Missingn* = *57 (4.4%)*					
Yes No	130 (11.9) 914 (83.9)	21 (20.0) 77 (73.3)	10 (14.9) 54 (80.6)	8 (34.8) 14 (60.9)	

Note. FSS = Functional Somatic Symptoms; FSS at T0 and T1 was dichotomized into high (top 10% score; high symptom load) and low scores (bottom 90% score; no/low symptom load) on basis of the entire participant population at the respective follow-up moment. Accordingly, FSS courses from pre- (age 11–12; T0) to late adolescence (age 16–17; T1) were categorized as following: No/Low FSS (bottom 90% FSS score at T0 and T1), Remittent FSS (top 10% FSS score only at T0), Incident FSS (top 10% FSS score only at T1) and Persistent FSS (top 10% FSS score at T0 and T1); * = Given regulations of data protection of the Denmark Statistics server, it is not allowed to present cell counts of > 0 but < 3 (i.e. microdata); ** = percentage is reported relative to FSS course group size

### Preadolescent FSS and FSS, anxiety and depression at late adolescence

In the current study, correlation between the assessment instruments were weak cross-sectionally and longitudinally for FSS and internalizing psychopathology at age 11–12 (*r* = .34), and for FSS at age 11–12 and anxiety (*r* = .21) and depression (*r* = .22) at age 16–17. Correlation between FSS at age 16–17 was moderate with anxiety (*r* = .64) and depression (*r* = .63) at the same time. Table 2 presents results from the multiple regression analyses of preadolescent (T0) FSS on square-root transformed FSS, anxiety and depression in late adolescence (T1). We found a statistically significant association for all respective outcome variables at T1, which remained significant when adding the different covariates. This suggests participants with higher self-reported FSS at the preadolescent age had increased self-reported scores on FSS, anxiety and depression in late adolescence compared to participants with lower self-reported preadolescent FSS even after controlling for sex, parental education, a chronic medical condition and internalizing psychopathology in preadolescence.

**Table 2:** Regression analysis summary for FSS at age 11–12 on FSS, anxiety and depression at age 16–17 (*N* = 1285)

	Outcome variable at T1	B	95% CI	SE	*p*	*n*
Model 1 (Crude)	FSS	0.07	0.06–0.08	0.01	< 0.001	1285
	Anxiety	0.05	0.04–0.07	0.01	< 0.001	1265; *Missing* 20 (1.6%)
	Depression	0.06	0.05–0.08	0.01	< 0.001	1262; *Missing* 23 (1.8%)
Model 2	FSS	0.06	0.05–0.07	0.01	< 0.001	1277; *Missing* 8 (0.6%)
	Anxiety	0.04	0.03–0.05	0.01	< 0.001	1257; *Missing* 28 (2.2%)
	Depression	0.05	0.04–0.07	0.01	< 0.001	1254; *Missing* 31 (2.4%)
Model 3	FSS	0.07	0.05–0.08	0.01	< 0.001	1222; *Missing* 63 (4.9%)
	Anxiety	0.04	0.03–0.05	0.01	< 0.001	1203; *Missing* 82 (6.4%)
	Depression	0.05	0.03–0.06	0.01	< 0.001	1201; *Missing* 84 (6.5%)
Model 4	FSS	0.06	0.04–0.07	0.01	< 0.001	1222; *Missing* 63 (4.9%)
	Anxiety	0.02	0.01–0.03	0.01	0.007	1203; *Missing* 82 (6.4%)
	Depression	0.03	0.01–0.05	0.01	< 0.001	1201; *Missing* 84 (6.5%)

Note. Square root transformation was used to transform respective outcome variables at T1 to meet assumption checks respective to linear regression models; FSS = Functional Somatic Symptoms; Models were composed with increasing adjustment as follows: (1) crude model, (2) sex, parental education (T0), (3) concurrent chronic medical condition (T0), (4) internalizing psychopathology (T0)

Based on the statistical effect estimates from Model 4, an example to visualize current findings could be an individual with a preadolescent FSS score of 40 (top 10% on the CSSI-24), who would score 1.80 points higher on square-root FSS (*95% CI* [1.20–2.10]), 0.60 points higher on square-root anxiety (*95% CI* [0.30–0.90]), and 0.90 points higher on square-root depression (*95% CI* [0.30–1.50]), at the late adolescent age than a participant scoring 10 (bottom 90% on the CSSI-24), given the same sex, sociodemographic background, presence of a chronic medical condition and score of internalizing psychopathology at age 11–12.

### Preadolescent FSS and FSS severity in late adolescence

Table 3 summarizes the results of the multinomial logistic regression analysis regarding the association between preadolescent FSS and FSS severity, i.e., (1) probable no to mild FSS, (2) probable moderate, single-organ FSS and (3) probable severe, multi-organ FSS in late adolescence. We found a significant relative risk ratio for FSS at T0 for both moderate and severe FSS severity at T1. For example, the risk ratio of a one unit difference in FSS scores in the probable moderate FSS group in late adolescence was 8% (*95% CI* [6 − 11%]) higher than the risk ratio of a 1 unit difference in FSS scores in the probable no/mild FSS group.

**Table 3:** Regression analysis summary for FSS at age 11–12 on FSS severity at age 16–17 (*N* = 1285)

FSS severity level (age 16–17)	RRR	95% CI	*p*
Probable no/mild FSS**			
Probable moderate FSS	1.08	1.06–1.11	< 0.001
Probable severe FSS	1.12	1.07–1.17	< 0.001

Note. FSS = Functional Somatic Symptoms; * = FSS severity level was based on a recent LCA (see Münker et al., 2022). Conditional probabilities of fulfilling specified symptom criteria were used, where we found a classification into class (1) probable no to mild FSS (i.e. a larger sub-group who did not report any significant physical symptoms in any symptom cluster), (2) probable moderate, single-organ FSS (i.e. a smaller sub-group who reported significant symptoms in one to two symptom clusters) and (3) probable severe, multi-organ FSS (i.e. the smallest sub-group who reported significant symptoms in multiple symptom clusters); ** = reference group, *RRR* = relative risk ratio

### FSS courses and internalizing psychopathology at late adolescence

Table 4 summarizes the results of the simple linear regression analysis on the association between the four FSS courses and anxiety and depression in late adolescence, with ‘no FSS at any time’ (i.e. no FSS at T0 and T1) as the reference group. There was a significant association between the FSS course and both anxiety and depression at age 16–17, indicating participants in all three FSS courses (i.e. incident, remittent and persistent) had significantly higher mean levels of anxiety and depression at late adolescence than those participants falling in the no or mild FSS at any time course. Moreover, based on the non-overlapping CI’s shown in Table 4, both the incident and persistent FSS courses had higher mean levels of anxiety and depression than the remittent FSS course, in comparison to no FSS at any time point.

**Table 4:** Regression analysis summary for FSS courses on square-root anxiety and depression at age 16–17 (*N* = 1285)

FSS courses	Outcome variable at T1	Mdn (Q1 – Q3)	B	95% CI	SE	*p*	*n*
No/Low FSS (Intercept)*	Anxiety	14 (8–22)	3.82				
	Depression	7 (3–14)	2.74				
Remittent FSS	Anxiety	19 (13–28)	0.58	0.29–0.86	0.14	< 0.001	1265; *Missing* 20 (1.6%)
	Depression	10 (6–23)	0.67	0.35–0.99	0.16	< 0.001	1262; *Missing* 23 (1.8%)
Incident FSS	Anxiety	34 (25–53)	2.20	1.85–2.54	0.18	< 0.001	1265; *Missing* 20 (1.6%)
	Depression	28 (16–38)	2.29	1.91–2.67	0.20	< 0.001	1262; *Missing* 23 (1.8%)
Persistent FSS	Anxiety	42.5 (31–57)	2.62	2.03–3.21	0.30	< 0.001	1265; *Missing* 20 (1.6%)
	Depression	28.5 (24–36)	2.71	2.05–3.37	0.33	< 0.001	1262; *Missing* 23 (1.8%)

Note. FSS = Functional Somatic Symptoms; T0 = pre-adolescence, age 11–12; FSS at T0 and T1 was dichotomized into high (top 10% score; high symptom load) and low scores (bottom 90% score; no/low symptom load) on basis of the entire participant population at the respective follow-up moment. Accordingly, FSS courses from pre- (age 11–12; T0) to late adolescence (age 16–17; T1) were categorized as following: no FSS at any time (no/low FSS at T0 and T1), remission (high FSS only at T0), incident (high FSS only at T1) and persistent (high FSS at T0 and T1); * = No/low FSS at any time was used as a reference group; the intercept represents the predicted mean of square-root anxiety and depression for the no/low FSS at any time group

## Discussion

### Main findings

In this population-based study of adolescents, high levels of self-reported FSS persisted in a small group over a period of 5 years. Socio-demographic differences were found across the FSS courses, with more females falling into the incident and persistent FSS course groups in comparison to reporting no FSS at neither time-point or only in preadolescence. Higher self-reported preadolescent FSS predicted higher levels of FSS and internalizing psychopathology in late adolescence. The association remained when we adjusted for socio-demographic variables, concurrent chronic somatic conditions as well as internalizing psychopathology in preadolescence. Furthermore, the risk for higher FSS scores at the preadolescent age was different according to the FSS severity group at late adolescence. Lastly, we found that all three specified FSS courses (i.e. incident, remittent and persistent) had significantly higher mean levels of anxiety and depression at late adolescence compared to the no or mild FSS at any time course, most pronounced in participants falling in the persistent and incident FSS courses.

### Comparison with previous literature and explanation for findings

Our finding of a persistent FSS course throughout adolescence corresponding to 1.8% is considerably lower compared to other studies investigating symptom continuity. Possible explanations for this discrepancy could be methodological differences regarding applied FSS measures, amount of assessment waves, study populations and age ranges of participants. For instance, approximately 14% of pediatric patients with functional abdominal pain from a clinical sample of six to 18 year old pediatric patients fell into a course characterized by persisting high symptom levels [13]. This is understandably a higher number compared to ours, as the current sample stems from a general population-based, non-clinical, cohort. Still, studies on general population samples have also reported higher numbers. In the Northern Swedish Cohort, a longitudinal cohort study on pupils in their last year of compulsory school, approximately 17% of participants fell into a high symptom course group from late adolescence to middle adulthood [14]. However, this difference may be explained by their much longer time span for follow-up (from ages 16–42 years). A Dutch study from Tracking Adolescents’ Individual Lives Survey (TRAILS) cohort is more comparable to our study in terms of the age range for follow-up [16], yet participants also reported more persisting symptoms throughout adolescence (i.e. 4.1% from age 11 to 16). However, their analysis was based on more assessment waves and using a different FSS measure (i.e., the Youth Self-Report and the Child Behavior Checklist). More similar findings to our study come from the Zurich Epidemiological Study of Child and Adolescent Psychopathology (ZESCAP), where FSS were persistently reported throughout three different assessment waves from late childhood to early adulthood by approximately 1 to 9% depending on the type of symptom, with the majority of individual persistent symptoms being below 4% [15]. Still, these results are not directly comparable to ours as we looked at a sum score and not developmental trajectories of single symptoms. Further, as we used different FSS measures at the two time-points in our study, meaning different symptoms as well as time frames for symptom presence were covered at preadolescence and late adolescence, this may have led to a potential underestimation of persistent FSS in our sample.

We found that FSS at preadolescence predicted internalizing psychopathology and FSS severity during later adolescence. Co-occurrence of FSS with anxiety and depression at and throughout various age stages have previously been reported [22–35, 37]. We provided further evidence that FSS in preadolescence predicted self-reported levels of anxiety and depression at late adolescence, even when accounting for increased internalizing psychopathology that already presented in preadolescence. Others have found that somatic symptoms at age 16–17 predict mental illnesses (i.e. mood disorders) in adulthood, while accounting for adolescent depression and anxiety [38]. In line with these findings, problems with emotion regulation and negative affect seem to play a prominent role in conditions characterized by somatic symptoms [75–77], also in childhood and adolescence [78–80]. The proposed emotional dysfunction ‘superspectrum’ as part of the Hierarchical Taxonomy of Psychopathology (HiTOP), a contemporary conceptual framework of psychopathology emphasizing associations between different types of symptomatology [48, 49], could be used to interpret current findings: The association between FSS and internalizing psychopathology might be explained through common risk factors and underlying dysfunctional processes, for instance in negative emotionality, cognitive difficulties (i.e. cognitive inflexibility) and/or childhood temperamental antecedents (i.e. low surgency with fearfulness, social withdrawal, behavioral avoidance) [50]. In addition, depression and somatic symptoms have been proposed to maintain and dynamically impact each other over time due to perpetuating cognitive-affective factors such as maladaptive emotion regulation strategies [81].

In accordance with this line of thought, our findings can further be interpreted within the concept of heterotypic psychopathological continuity [82–84]. We potentially found evidence for both a homotypic (i.e. preadolescent FSS predicted FSS and associated symptom severity at late adolescence) as well as heterotypic (i.e. preadolescent FSS predicted anxiety and depression at late adolescence) continuity from pre- to late adolescence in the current study. Participants falling into both the incident and persistent but also the remittent FSS course reported higher mean levels of anxiety and depression compared to those in the no or mild FSS course. This may suggest, that even though FSS often remit and are self-limiting during adolescence, they can still pose a risk for elevated anxiety and depression later on, as also shown in related literature [24, 29, 38–40]. The question remains whether this increased risk is simply due to high preadolescent FSS. Thus, it is important to note that we cannot conclude about a longitudinal association between the FSS courses throughout adolescence and anxiety and depression in late adolescence as the course variable also included FSS at age 16/17. Therefore, the association could simply be explained by a high FSS score in late adolescence co-occurring together with higher levels of anxiety and depression, rather than representing a longitudinal association with persisting symptoms throughout adolescence. However, on basis of current findings, we suggest that high levels of self-reported FSS at either time point, in pre- or late adolescence, could have adverse outcomes for internalizing psychopathology in late adolescence. Nonetheless, it is important to note that in the sense of homo- and heterotypic patterns, we only considered internalizing psychopathology in this study, and hence cannot conclude on heterotypic patterns across more diverse psychopathological domains.

In sum, we discussed our findings according to current conceptualizations of psychopathology (i.e. The HiTOP model of psychopathology dimensions, homotypic vs. heterotypic continuity and mutual maintenance) and interpret our findings as evidence for co-occurrence of FSS, anxiety and depression over time. However, it is important to note that the associations between these symptom dimensions were rather weak, as preadolescent FSS predicted small differences on all outcome values in late adolescence. We therefore emphasize the need for further longitudinal research to investigate the dynamic interplay and the clinical relevance of the association between FSS and internalizing psychopathology, with potential mutually triggering as well as perpetuating factors in early life stages.

### Strengths and limitations

The current study employed data from a general population-based child cohort as well as Danish national registers with a substantial sample size [85, 86]. Furthermore, we applied validated instruments for the assessment of variables of interest. Nonetheless, some limitations should be considered. First, there was attrition from the original CCC2000 sample, where participants with complete FSS data at 11–12 and those with follow-up data at age 16–17 were more often female and had parents with higher levels of education, compared to non-participants. However, participants with follow-up data at age 16–17 did not differ significantly from non-participants on their baseline measures of FSS, internalizing psychopathology and presence of a chronic medical condition at age 11–12. This implies that those lost to follow-up at age 16–17 did not experience significantly more somatic symptoms, emotional problems or suffered from a chronic medical condition in preadolescence compared to those with complete FSS data at both assessment waves. Nonetheless, differences on sociodemographic background variables likely reduce variability in participant’s characteristics due to positive selection. This could lead to biased conclusions in the direction of reporting more attenuated results (i.e. less “extreme” responses) [87]. Overall, the findings might not be generalizable to less advantaged populations. Second, it is possible that the current sample is biased simply by already having been part of this cohort study over various follow-up waves. Participants might be more aware about for instance their own mental health status through having been assessed repeatedly over time in comparison to the general population. In turn, their responses might not reflect those of the general population anymore.

Third, another limitation was that different measures were used to assess FSS at the two time-points, which could impact the comparison of the two FSS assessments and thereby also the estimation of the different FSS courses. Fourth, adjusting for internalizing psychopathology at age 11–12 in the final model on the associations between preadolescent FSS and the various outcomes in late adolescence might be considered as ‘over-adjusted’, i.e. adjusting for potentially overlapping constructs and thereby attenuating the estimates [88]. Fifth, the results on the association between preadolescent FSS and FSS severity at late adolescence should be interpreted with caution due to low statistical power. This study adds to the currently scarce scientific literature in the field, and was therefore primarily explorative, using a quite simple statistical approach based on only two assessment points to explore the research questions. Future studies could extend the current findings by longitudinal study designs with multiple assessment points and the use of more sophisticated statistical models to understand the temporal associations between FSS, anxiety and depression in more depth.

## Conclusion

This study contributed to the still scarce scientific evidence on the course of FSS and associations between FSS and internalizing psychopathology throughout adolescence. In the context of current conceptual frameworks for psychopathology that aims to integrate and understand overlapping psychopathology, we suggest that shared mechanisms might underlie FSS, anxiety and depression. We recommend health care professionals to be attentive of these co-occurring and interacting symptom dimensions and the potential adverse impact of high FSS in preadolescence on later psychological functioning in their clinical assessment and treatment approaches.

### Key points

- Functional Somatic symptoms (FSS) are common already in childhood and adolescence. Over time, they are associated with internalizing psychopathology such as anxiety and depression.
- This study examines FSS courses from pre- to late adolescence and the association between preadolescent FSS and internalizing psychopathology in late adolescence in a population-based birth cohort.
- The majority reported no FSS at either time point in adolescence, with more adolescents falling into a remitting than incident FSS course group, and a smaller group with persistent FSS.
- Higher self-reported preadolescent FSS predicted FSS, anxiety and depression at late adolescence.
- Additional longitudinal research is needed to determine how FSS and internalizing psychopathology relate to each other dynamically over time, including shared vulnerability factors of these co-occurring symptom expressions.

## Electronic supplementary material

Below is the link to the electronic supplementary material.

Supplementary Material 1

Supplementary Material 2

## Data Availability

We used sensitive personal data which cannot be shared publicly due to data protection laws in Denmark by the Danish Data Protection Agency. All data was pseudonymized and stored on a digital server of Statistics Denmark. Any researcher can receive data access under the same regulation as the authors (access granted case-by-case through approval from the CCC2000 steering committee having legal responsibility as data manager). Access will be granted to a permissible extent by the General Data Protection Regulation (GDPR) and the Danish Data Protection Act. If access is granted, the PI investigator and last author (Charlotte Ulrikka Rask, charrask@rm.dk) will make data available with approved authority to access pseudonymized data through Statistics Denmark. The General Data Protection Regulation (GDPR) and the Danish Data Protection Act and regulations do not allow any other forms of data sharing.

## Abbreviations

FSS: Functional Somatic Symptoms
