# Supplementary material for: Exploring the course of functional somatic symptoms (FSS) from pre- to late adolescence and associated internalizing psychopathology – an observational cohort-study
Source: BMC Psychiatry. 2024 Jul 8;24:495. doi: 10.1186/s12888-024-05937-3 (PMC11232134; doi:10.1186/s12888-024-05937-3)
Supplement: Supplementary file 1 — Supplementary Material 1 [file 12888_2024_5937_MOESM1_ESM.docx]

Appendices

*Appendix 1: Attrition analyses, comparing participants with follow-up data on FSS at age 16-17 to those lost to follow-up*

| Variables | Follow-up at 16-17  (N = 1285)  Frequency *n* (%) | | Lost to follow-up after 11-12  (*n* = 605)  Frequency *n* (%) | Statistics |
| --- | --- | --- | --- | --- |
| Sex  Male  Female | 573 (44.59)  712 (55.41) | | 325 (53.72)  280 (46.28) | χ2^a^ = 13.74, df(1), *p* < .001* |
| Parental education level  Primary school education  Short Traineeship | 67 (5.21)  865 (67.32) | | 72 (11.90) 407 (67.27) | χ2^a^ = 35.03, df(2), *p* < .001* |
| Long Traineeship/  University education | 345 (26.85) | | 116 (19.17) |  |
| *Missing* | *8 (0.62)* | | *10 (1.65)* |  |
|  | Frequency *n* (%)  *M(SD); Mdn (Q1 – Q3)* | Frequency *n* (%)  *M(SD); Mdn (Q1 – Q3)* | |  |
| FSS at age 11-12 | 1285 (100)  5.45(6.10); 4 (1 - 7) | | 605 (100)  5.09(5.67); 3 (1 - 7) | *z^b^*= -1.514, *p* = 0.130 |
| Internalizing psychopathology at age 11-12 | 1285 (100)  1.72(1.72); 1 (0 - 3) | | 605 (100)  1.70(1.73); 1 (0 - 3) | *z^b^* = -0.406, *p* = 0.684 |
| Presence of a chronic medical condition at age 11-12 | 1228 (95.56) | | 518 (85.62) | *z^b^* = -1.681, *p* = 0.092 |
| Missing | 0.14(0.34); 0 (0 - 0)  *57 (4.44)* | | 0.11(0.31); 0 (0 - 0)  *87 (14.38)* |  |

*Note*. * = significant at inference level α = .05; ^a^ = Chi-square test; ^b^ = Two-sample Wilcoxon rank-sum (Mann–Whitney)
