# Supplementary material for: Exploring the course of functional somatic symptoms (FSS) from pre- to late adolescence and associated internalizing psychopathology – an observational cohort-study
Source: BMC Psychiatry. 2024 Jul 8;24:495. doi: 10.1186/s12888-024-05937-3 (PMC11232134; doi:10.1186/s12888-024-05937-3)
Supplement: Supplementary file 2 — Supplementary Material 2 [file 12888_2024_5937_MOESM2_ESM.pdf]

Professor Robert Goodman support@youthinmind.com
Youthinmind Ltd
114 Court Lane
Dulwich Village
London SE21 7EA
Company Registration No: 4044574
VAT No: 980 6486 81

youth*in*mind

24 June 2024

To: **BMC Psychiatry**

Pia Jeppesen, Copenhagen Child Cohort 2000 (CCC2000) studies, used an authorised version of the Development and Well-Being Assessment (DAWBA) and the Strengths & Difficulties Questionnaire (SDQ) in accordance with the copyright, and we give Pia Jeppesen permission to publish this paper.

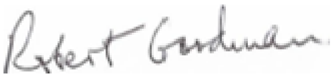

Professor Robert Goodman, PhD, FRCPsych, MRCP
Director
